# Coagulation abnormalities and vascular complications are common in PGM1-CDG

**DOI:** 10.1016/j.ymgme.2024.108530

**Published:** 2024-07-02

**Authors:** Silvia Radenkovic, Sofie Bleukx, Nicole Engelhardt, Erik Eklund, Saadet Mercimek-Andrews, Andrew C. Edmondson, Eva Morava

**Affiliations:** a Department of Clinical Genomics, Mayo Clinic, Rochester, MN, USA; b Department of Genetics, Section Metabolic Diagnostics, UMC Utrecht, Utrecht, NL; c Division of Human Genetics, Department of Pediatrics, Children’s Hospital of Philadelphia, PA, USA; d Departement of Clinical Sciences, Lund, Pediatrics, Lund University, Lund, Sweden; e Nuffield Department of Clinical Neurosciences, University of Oxford, Oxford, UK; f Department of Medical Genetics, Faculty of Medicine and Dentistry, University of Alberta, Edmonton, Alberta, Canada; g Department of Genetics and Genomics Sciences, Icahn School of Medicine at Mount Sinai, New York, NY, USA; h Department of Biophysics, University of Pécs Medical School, 7624 Pécs, Hungary

**Keywords:** Phosphoglucomutase-1, Congenital disorder(s) of glycosylation, Coagulation abnormalities, Galactose therapy

## Abstract

Phosphoglucomutase-1-congenital disorder of glycosylation (PGM1-CDG) is a rare genetic disorder caused by biallelic variants in the *PGM1* gene, leading to the deficiency of the PGM1 enzyme. The most common clinical presentations include muscle involvement, failure to thrive, cleft palate, and cardiac involvement. Abnormal serum N-glycosylation, hypoglycemia, and liver function abnormalities including coagulation abnormalities are the most common laboratory abnormalities. While PGM1-CDG has been extensively studied, little is known about the extent of the coagulation abnormalities in individuals with PGM1-CDG. Unlike most CDG, some symptoms of PGM1-CDG are treatable with D-galactose (D-gal) supplementation, though reliable clinical endpoints are necessary to appropriately evaluate the potential improvement with D-gal in PGM1-CDG. Here, we aimed to describe the incidence of coagulation abnormalities in PGM1-CDG and their evolution, their relation to clinical events, and the ability of D-gal treatment to improve them. A retrospective analysis was conducted on 73 reported individuals. All individuals had a molecularly confirmed PGM1-CDG diagnosis. All incidences of antithrombin (AT), aPTT, PT, factor (F) XI, FX, FIX, FVII, protein C and protein S data and major clinical events related to coagulation abnormalities, were collected. Coagulation information was available for only 58.9 % of the reported individuals, out of which 67.4 % of PGM1-CDG individuals were reported to have abnormalities. The most frequently observed abnormality was AT (mean: 30.8% R:80–120 %) deficiency. Four individuals had major thrombotic events. Coagulation status on D-gal treatment, were reported in 19 individuals. Several factors showed improvement including AT (mean: 64.5 %), indicating galactose is beneficial in treating coagulation abnormalities in PGM1-CDG. Due to the scarcity of the reported data on coagulation parameters, we also evaluated data collected in sixteen PGM1-CDG individuals enrolled in the FCDGC Natural History Study. Longitudinal data showed improvements in several coagulant parameters and disease severity improved for almost all patients of whom we had multiple datapoints on D-gal. AT showed significant improvement on D-gal. We conclude that coagulation abnormalities are frequently present in PGM1-CDG and show improvement on D-gal. We recommend coagulation parameters should be routinely checked in individuals with PGM1-CDG or suspected of having PGM1-CDG. Finally, AT may be used as a primary or secondary clinical endpoint for upcoming clinical trials in PGM1-CDG individuals.

## 1. Introduction

Congenital disorders of glycosylation (CDG) are a group of inborn errors of metabolism that are caused by defects in the glycosylation [1,2]. CDG are caused by pathogenic variants in enzymes located in the cytosol, ER or GA. CDG can affect all types of glycosylation including N-glycosylation, O-glycosylation and GPI-anchor synthesis defects. CDG affecting N-linked glycosylation are usually classified into two types-CDG-I and CDG-II. When a CDG defect results in the abnormal N-glycosylation in the ER, it is classified as a CDG—I, which usually results in complete loss of glycan chains. CDG-II are caused by a defect in N-glycosylation in the Golgi apparatus (GA) which usually leads to truncated glycans [3] [4]. Abnormalities in N-glycosylation are usually diagnosed by assessing patterns in carbohydrate deficient transferrin (CDT) in blood to determine whether the individual is affected by CDG-I or CDG-II. This can be done either by transferrin isoelectric focusing (TIEF) or by different methods based on mass-spectrometry (MS) [5,10].

Phosphoglucomutase-1 (PGM1) deficiency, previously named glycogen storage disease XIV, is also classified as a CDG (PGM1-CDG, OMIM: 614921) [6,7]. The PGM1 enzyme, which is located in the cytosol, is responsible for the conversion of glucose-1-phosphate into glucose-6-phosphate [8] and its deficiency affects glycolysis, glycogen metabolism and glycosylation [7]. PGM1-CDG is a unique CDG as it presents with a combined CDG-I and CDG-II TIEF pattern, which is diagnostic and helps us distinguish PGM1-CDG from other types of CDG [7].

Individuals with PGM1-CDG usually have a multisystem presentation which includes muscle and heart involvement, liver involvement, congenital malformations, and endocrine and hematological abnormalities [9,10].

As coagulation factors are heavily glycosylated, it is no surprise that many CDG, including PGM1-CDG, also present with coagulation abnormalities. As these hematologic abnormalities can lead to an increased risk of thrombotic events and bleeding, the recommendation is to perform a coagulation assessment every year and prior to surgery [10].

Abnormalities in both pro- and anticoagulant factors have been reported in PGM1-CDG. For example, antithrombin III (AT), factors (F) VII, IX, X XI, along with activated partial thromboplastin time (aPTT) and prothrombin time (PT) can be affected. Nevertheless, patients can present with different degree of coagulation abnormalities, which is not completely understood. Therefore, understanding the disease variability in PGM1-CDG is crucial to improve clinical care [9].

In addition to its unique biochemical presentation, PGM1-CDG also stands out as one of the few CDG for which a treatment option exists: D-galactose (D-gal) [10–16] therapy. D-gal, a naturally available sugar, was proposed as a treatment for PGM1-CDG based on the presence of hypogalactosylated glycans in blood; however, the exact therapeutic mechanism was not clear [11]. Previously, it was shown that PGM1-CDG presents with a decrease in the glycosylation nucleotide sugar donors UDP-glucose and UDP-galactose, which are replenished by D-gal [17]. As UDP-glucose is used to make growing glycan chains in the Endoplasmic Reticulum (ER) and UDP-galactose is necessary for the part of glycosylation that occurs in the Golgi apparatus (GA), these findings also explained the presence of the mixed CDG type in PGM1-CDG [11]. D-gal has been shown to improve various aspects of PGM1-CDG such as liver function [7,16–18]. Also, some individuals have experienced improvement in coagulation, exercise tolerance, fatigability, had fewer bouts of rhabdomyolysis, and entered puberty [7,16–20].

Though D-gal has been trialed in several patients [11–13,16,21,22], no formal clinical trial has been initiated so far due to the lack of clinical endpoints and a natural history study in PGM1-CDG. Specifically, the data regarding the positive effects of D-gal therapy on coagulation is sparse.

In general, hemostatic abnormalities are not frequently assessed in CDG, thought they have previously been reported in PMM2-CDG [24,46] In addition, it has been shown that coagulation abnormalities frequently correspond to the acute complications in PMM2-CDG, warranting studies in other CDG [24].

To further understand the natural history of PGM1-CDG and the effect of D-gal therapy on coagulation factors, we have performed a retrospective study on reported PGM1-CDG individuals. In addition, we analyzed data collected in PGM1-CDG individuals currently participating in Frontiers in Congenital Disorders of Glycosylation (FCDGC) Natural History study (Clinical and Basic Investigations into Congenital Disorders of Glycosylation ClinicalTrials.gov ID: NCT04199000). We collected data on AT, FVII, FIX, FX, FXI, aPTT and PT, protein S and protein C. In addition, we collected phenotype severity information [23] and clinically significant coagulation events on each patient when available.

Our findings provide insight into the natural incidence and evolution of coagulation abnormalities in PGM1-CDG and the ability of D-gal to improve them. We show that the coagulation factors could be used to predict and evaluate the effects of D-gal therapy or other potential new treatments in upcoming clinical trials.

## 2. Materials and methods

### 2.1. Literature search

PubMed database was used to perform a systematic literature review using the following key words: “phosphoglucomutase-1 deficiency”, “PGM1-CDG”, “glycogen storage disorder type XIV”, “CDG type It” which identified 22 articles published between 2008 and 2023.

### 2.2. Subjects

Patients with a confirmed molecular diagnosis of PGM1-CDG were included in this study. Retrospective data was collected from 73 reported PGM1-CDG individuals identified by the literature search. Natural history data analysis was performed in 16 individuals, 9 of which were reported (P14, P67-P73). All PGM1-CDG individuals whose data were longitudinally collected were enrolled in the Frontiers in Congenital Disorders of Glycosylation Natural History Study (IRB: 19–005187). Informed consent was collected for all the recruited patients. Demographic information of all the individuals is available in the supplementary file.

### 2.3. Data extraction

Demographic information was collected for all individuals. For the reported individuals, the age of the individual at the time of the publication was recorded. For those followed longitudinally (retro- and prospectively), the age at the most recent visit was recorded. Clinically relevant information related to coagulation including antithrombin levels (AT), aPPT, PT, FVII, FIX, FX, FXI, protein C and protein S activity were collected. Platelet count was collected when available. Major clinical events such as bleeding episodes, strokes, and thrombotic events were also recorded. Apart from the data related to coagulation, Nijmegen Pediatric CDG Rating Scale (NPCRS) scores (phenotype severity scores) were collected if available [25,26].

### 2.4. Statistical analysis

Welch *t*-test was performed to assess the effect of D-gal on different coagulation parameters. However, statistical analysis was not possible in several cases due to the lack of datapoints.

## 3. Results

### 3.1. Clinical and demographic information of the reported PGM1-CDG individuals included in the study

To assess coagulation abnormalities in PGM1-CDG, we began by assessing 73 reported PGM1-CDG individuals published between 2008 and the end of 2023. Demographic information from the reported PGM1-CDG individuals included in this study is available in Supplementary Materials (Supplementary Table 1). The ages, as reported in original publication, ranged from four months to 53 years and included 60.2 % male and 39.7 % female individuals. Of the available information regarding self-reported race, the majority identified as white. All the individuals had molecularly confirmed variants in *PGM1*. The majority of the individuals were compound heterozygous, with the most common pathogenic variant being c.112 A > T (9 individuals). Complete demographic and genetic information for all the reported individuals can be found in Supplementary Table 1.

The coagulation status of the reported individuals was available for 43/73 (58.9 %), out of which 29/43 individuals (67.4 %) had coagulation abnormalities (Table 1). Very limited data was available for the majority of the coagulation parameters. For several individuals, exact values for specific coagulation parameters were not reported and the only information available was whether the parameter was ‘normal’, or ‘abnormal’, or, in the case of aPTT and PT, ‘normal’ or ‘delayed’. The most commonly evaluated coagulation parameters were aPTT and AT, followed by PT, FIX, and FXI. Protein S and protein C activity were reported in 3 individuals, and there was 1 individual with values reported for PTT (which is a test similar to aPTT), FVII, and FX (P21) (Table 1).

The AT levels were abnormal in 9/9 individuals (mean values = 30.8 %, reference range (R): 80–130 %). The aPTT values were reported in 11 individuals. In in 5 individuals, aPTT was reported as decreased, without reporting the values. Out of the other 6 individuals with reported values, 3 were abnormal (mea*n* = 41 s, R:24–45 s). PTT was assessed in 1 individual, who had abnormal values (41.8 s, reference range: 18–28 s). The PT levels were abnormal in 5/6 individuals. FIX values were normal in all 6 evaluated individuals at baseline. FXI values were decreased in 4/4 individuals (mea*n* = 30.5 %, R: 70–120 %). Protein C and protein S activity were reported in 2 individuals and both had abnormal values. FX and FVII were abnormal in the same individual. Detailed information on the coagulation parameters collected from reported initials without D-gal therapy can be found in Table 1 and Supplementary Table 1.

From 73 reported individuals [10–16,22,27–40], only 19 had been trialed on D-gal treatment (P5, P14, P30, P32, P34, P44, P45, P48, P49, P50, P51, P56, P67-P73) (Supplementary table 1).

The most commonly evaluated parameters on D-gal were ATand FIX respectively. After starting D-gal, AT levels improved, however, they were still abnormal in 5/7 individuals (mea*n* = 64.5 %; R: 80–130 %). FIX levels remained in the normal range in all 5 individuals whose levels were assessed on galactose treatment. FXI was abnormal in 2/2 individuals, however, it normalized on long-term D-gal treatment (>18 weeks). PT was slightly abnormal in 1/1 individual and aPTT in 1/2 individuals who received D-gal. PTT, which No protein C or protein S activity, FVII or FX values were reported on D-gal treatment. The information available on coagulation markers after the initiation of D-gal therapy is listed in Table 1. Detailed information of the laboratory results can be found in Supplementary Table 1.

In addition to coagulation parameters, we have collected the information on major vascular events in reported PGM1-CDG individuals. A vascular event was reported in 4 individuals (P2, P8, P29, P69) [11,16,28,33,34]. The most severe vascular event involved an 8-year-old male who died due to a cerebrovascular thrombosis (P2) [11,28,34]. Thrombosis was reported in a further 3 individuals, one of who experienced repeated episodes of thrombosis (P8) [11] and one that had thrombosis of the left internal carotid artery (P29) [33]. One individual (P69) had deep vein thrombosis (DVT). All of the individuals who experiences major vascular events were reported to have coagulation abnormalities (Supplementary Table 1), however, no coagulation parameters values were available for P2 and P8. P29 had abnormal, AT, protein C and protein S activity levels (around 50 %) [7,41] and in P69 prolonged aPTT was recorded [42].

### 3.2. Longitudinal analysis of coagulation factors in PGM1-CDG individuals enrolled in the FCDGC natural history study

Previous reports had a general lack of datapoints that would allow us to gain insight into the natural history of coagulation in PGM1-CDG and assess the effect of the long-term D-gal treatment on coagulation factors. To further understand this, we performed a longitudinal (retro and prospective) analysis in the 16 individuals that were enrolled in the FCDGC natural history study or clinically followed by our collaborators (P67-P82). From these individuals, eight were reported (P14, P67–73) [11,15,16,34]. D-gal treatment was started in 15/16 individuals. Coagulation parameters for three individuals (P67, P70, P81) were not available prior to starting D-gal. The demographic information of the 16 individuals as well as the values of different coagulation parameters and other clinical data that was collected are provided in the Supplementary materials.

**Fig. 1.**
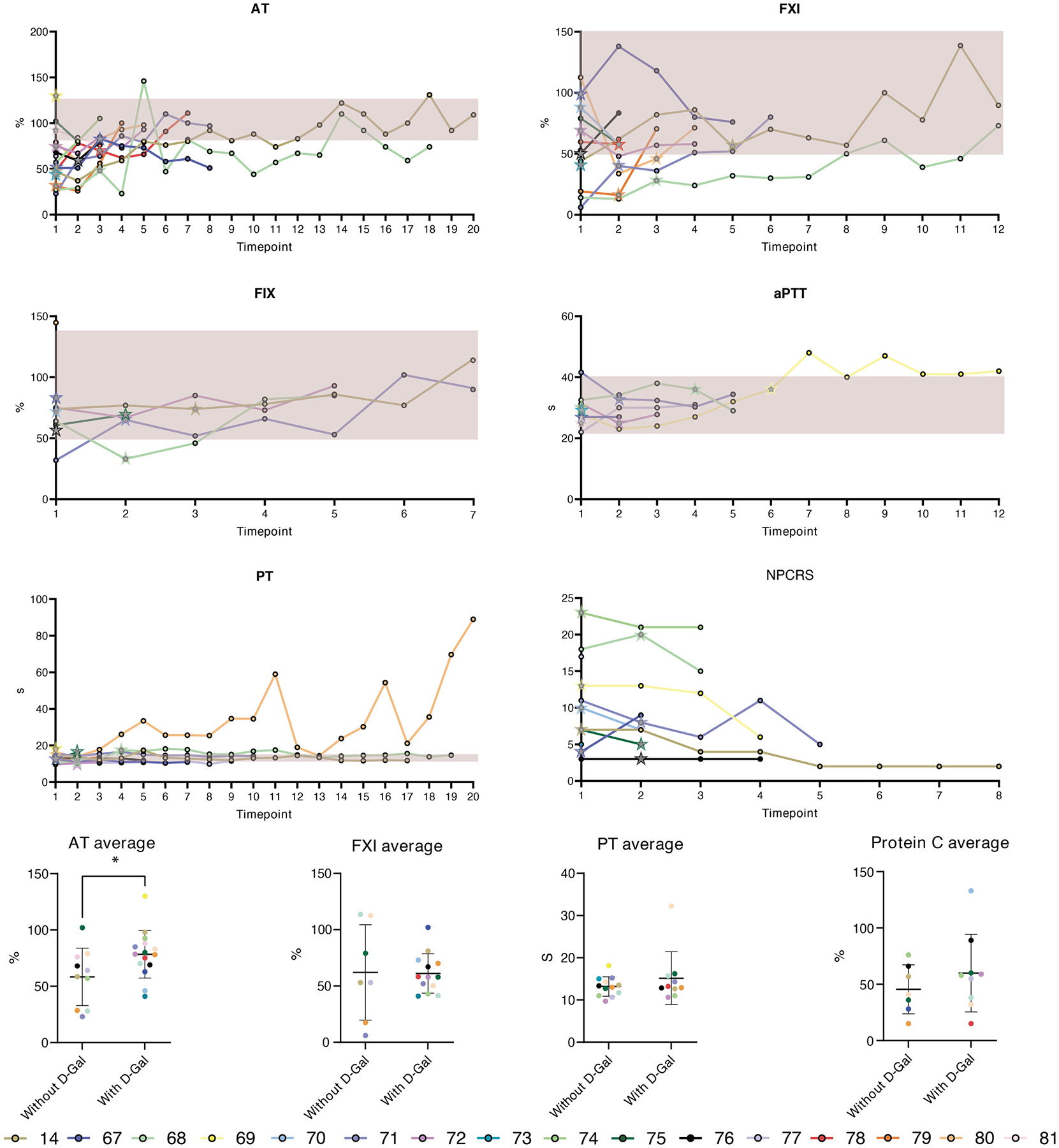
Long-term evaluation of the major coagulation parameters in PGM1-CDG individuals. AT, aPTT, PT, Factor IX and Factor XI were assessed. The majority of the individuals included in our longitudinal study received galactose therapy. The start of the D-gal therapy is indicated by a star in the graph. The most common reference range is indicated in light orange. Note: the individuals were evaluated in different laboratories, which report different reference ranges. Details of the reference ranges and reported values used to make above shown graphs are listed in the supplementary table. Numbers in the legend refer to the individual ID number assigned in Supplementary Table 3. Time points indicate different times the patients had their coagulation parameters evaluated and are not linear. The exact dates of each measurement are given in the Supplementary Table. *P80 suffered from warfarin toxicity, resulting in extreme PT values.

We compared coagulation abnormalities before and after D-gal treatment, when available, in this group. The mean AT levels in the individuals without D-gal was 60.83 %, while the mean AT levels on D-gal were 78,7% (most commonly used R: 87–145 %). On D-gal, normal levels of AT were reported at least once in 7/8 individuals. However, in some of the patients the values of AT still fluctuated on therapy (Table 2, Fig. 1). The average time needed to reach the first normal AT value the individuals was 4–5 months after starting D-gal therapy, while improvement was seen as soon as two months after initiating therapy.

In our individuals, FIX was typically within the normal range. One individual (P71), had abnormal FIX before starting D-gal which normalized after 2 years of therapy (P71). The mean levels of Factor IX were 74 % without D-gal and 71 % on D-gal (R: 50–150 %). FX was not commonly assessed in our cohort and was within the normal range in all the individuals who had FX assessed (P14, P75, P79).

FXI was abnormal in 4/7 individuals. The average FXI levels were 48 % (R: 63–142 %). On D-gal, the average FXI was 61 % and it reached normal values in 3 individuals (P71, P76, P79). One individual (P14) had normalization on lactose prior to starting D-gal. The time to the first normal result spanned between 3 months to 2 years after initiating D-gal therapy. FVII and FX were the least frequently assessed parameters in our PGM1-CDG cohort. FVII was abnormal in 2/4 individuals without D-gal (P14, P75, P79, P80) and in one patient on D-gal (P68). FX was normal in 4/4 assessed individuals without D-gal, and abnormal in 1 individual (P74) and normal in the other individual (P75) whose FX was assessed on D-gal (Table 2).

On the other hand, protein C was assessed in 10 individuals, out of which 9 had abnormal values (mean = 56 %). There was no difference in the mean of protein C on D-gal. However, protein C normalized in 2 individuals (P68, P75) on D-gal, while protein C activity normalized on lactose in the individual that received lactose supplementation prior to starting D-gal (P14).

PT was abnormal in 5 individuals (P68, P71, P76, P14, P80) and normalized in 2 individuals on D-gal (P71, P76). One individual (P14) had fluctuating PT levels both with and without receiving D-gal, while P81 had severely abnormal PT throughout the course of D-gal treatment. aPTT was slightly outside the normal range in 3 out of 10 individuals (P68, P69, P71) prior to starting D-gal. This normalized within the first year of starting D-gal in P71, while aPTT remained mostly abnormal in P68. aPTT increased and remained abnormal in one individual after starting D-gal (P69). PTT, which measures the same clotting abnormalities as aPTT, was measured in 8 patients, out of which 4 (P14, P75, P77, P80) reported prolonged PTT prior to starting D-gal (Table 2, Sup Table 3). After starting D-gal, PTT was prolonged in two individuals (P68, P80), but normalized after 30 months (P68) and 5 months (P80) on D-gal (Table 2, Sup Table 3).

As the available data regarding the coagulation parameters was scarce across reported PGM1-CDG individuals, we aimed to collect more information on the natural history and beneficial effects of D-gal by assessing the phenotype severity scores (NPCRS) [23,34]. A higher severity score typically indicates a more severe phenotype, where a lower score represents a milder phenotype. Severity scores are divided into sections (I, II, III), each evaluating different aspects of the phenotype.

NPCRS scores were used to assess disease severity and beneficial effects of D-gal. NPCRS improved in 7/10 individuals receiving D-gal, showing overall decrease of phenotype severity.

Out of 16 patients enrolled in FCDGC Natural History study, two reported major vascular events (P69, P80). One of the patients (P69) experienced an episode of deep vein thrombosis (DVT), while the other patient (P80) had an ischemic stroke with an acute left middle cerebral artery (MCA) infarction. In addition, one patient (P77) had a cardiac arrest during ear tube placement. No data was available for the coagulation parameters at the time of the cardiac arrest. P69 had prolonged aPTT around the time the DVT was reported and P80 had slightly decreased PTT time at the time of stroke. No other coagulation parameters were assessed for these patients. None of the patients were undergoing D-gal therapy at the time of these events. On D-Gal, three patients (P69, P71, P72) reported easy bruising and/or (nose) bleeding.

Information of the evolution of coagulation markers after the treatment are listed in Table 2. All the datapoints collected throughout the natural history are shown in Fig. 1. Detailed information is given in the Supplementary Table 3.

## 4. Discussion

Pro-coagulant factors and physiological coagulation inhibitors factors are crucial for the regulation of bleeding and clotting of the blood. Therefore, abnormalities in coagulation can be life threatening. For example, the deficiency in pro-coagulation factors such as FVIII or FIX can result in severe bleeding, such as seen in hemophilia. On the other, a deficiency in anticoagulation factors can lead to thrombosis and is usually associated with life-threatening events. For example, deficiency of AT, a key endogenous coagulation inhibitor, can be especially severe [43].

Most of the coagulation factors are glycosylated, and abnormal coagulation parameters such as decreased AT have been reported in several CDG [10,24,44–48]. Nevertheless, the natural history of coagulation abnormalities in CDG is understudied and the clinical management of coagulopathies in CDG is further challenged by the fact that both pro- and anticoagulant factors can be affected in these disorders. Previous research [24,44,46] which aimed to assess the impact of combined pro- and anti-coagulant factor deficiencies on the hemostatic balance in CDG patients suggested that thrombotic events in CDG individuals can occur when the deficiency of anticoagulation factors is greater than that of the pro-coagulation factors. While AT deficiency has been reported in several CDG patients [44–46], pro-coagulation factors have not been extensively studied. In addition, the exact incidence of thrombotic events or other major vascular events in CDG is not known. As coagulation abnormalities can lead to life-threatening events, there is a crucial need for deeper understanding of the coagulation abnormalities in individuals with CDG to improve clinical care. Therefore, the existence of emergency protocols, especially in the absence of treatment in CDG (such as D-gal) is crucial. The emergency protocols for PMM2-CDG have already been described [24] in great detail, which can also be useful for other CDG, such as PGM1-CDG.

Previously, abnormalities in several coagulation parameters were shown PMM2-CDG [24,46]. Moreover, the link between hemostasis, neurological episodes, and fever, which further disrupt acute episodes in PMM2-CDG were demonstrated, highlighting the need to investigate hemostatic abnormalities in other CDG. For example, little is known about the natural history of coagulation abnormalities in other CDG such as PGM1-CDG. Moreover, though D-gal therapy is used to correct glycosylation abnormalities in PGM1-CDG [11–16,21,22], its beneficial effect on different coagulation parameters is not clear.

In our study, we found that only 58,9% of the reported PGM1-CDG individuals had information regarding coagulation of which 67,4% reported abnormalities (Table 1). The most commonly reported coagulation parameter was AT (*n* = 10). AT levels were reported in 9 patients without D-gal therapy, all of which were abnormal (Table 1). Abnormalities in FXI, FX, aPTT and PT were also frequently reported. Interestingly, though protein C activity was only assessed in three PGM1-CDG individuals and was abnormal in all three (100 %) (Table 1). Whether it was assessed and found normal in others is not known.

Major thrombotic events were reported in 4 individuals (P2, P8, P29, P69) [11,16,28,33,34]. No information regarding the coagulation factor values was available for P2, P8. P29 at the time of the event had decreased AT, protein C and protein S activity values around 50% [33], while P69 had prolonged aPTT [16]. None of the individuals were receiving D-gal at the time of the event. It is interesting to note that both venous and arterial events occurred, whereas in PMM2-CDG, the thrombotic events are on the venous side only [45,46].

As the data regarding the coagulation parameters in reported PGM1-CDG individuals was scarce, we then analyzed the coagulation parameter data of the individuals participating in the FCDGC Natural History Study. We also evaluated the effect of D-gal therapy on coagulation parameters in the prospectively followed individuals (Table 2, Fig. 1). AT was the most frequently measured coagulation parameter and also the most frequently abnormal. Though there were fewer measurements available for FXI, FIX, and protein C, these parameters were also frequently abnormal in our cohort (Table 2). PT, aPTT and PTT were also frequently assessed (Table 2), though their values over time did not drastically change regardless of D-gal treatment (Fig. 1). On the other hand, FVII and FX were not as frequently assessed, however, both abnormal and normal values were recorded with and without D-gal (Table 2, Sup Table 3). As the number of individuals who had FVII and FX assessed was low, at this time we are unable to draw any conclusions to whether they are also frequently abnormal in PGM1-CDG and what the D-gal effect on these factors is. Therefore, there is a crucial need to systematically collect data on these factors in other PGM1-CDG individuals as well, in order to better understand hemostatic abnormalities caused by PGM1 deficiency.

There was also a variability in the values of all the coagulation parameters, even within the same individual, when followed overtime. Nevertheless, we found that D-gal resulted in improvement of both pro- and anti-coagulation factors (Table 2). In addition, the longitudinal data showed a decrease of NPCRS scores for all but one patient on D-gal therapy (Fig. 1), confirming previous observations that D-gal improves overall severity of PGM1-CDG [10,13,14].

To assess the utility of different coagulation parameters as a potential clinical end point for future trials with D-gal or other potential treatment options, we compared the average values of different coagulation parameters with or without D-gal (Fig. 1). This was quite challenging as several of the coagulation parameters were not checked regularly and moreover, many coagulation parameters levels fluctuated on D-gal before stabilizing. In addition, more data was available for coagulation parameters during D-gal treatment, likely due to individuals undergoing D-gal being more closely monitored.

We found that there was a statistically significant improvement in the average levels of AT with D-gal compared to untreated individuals in our cohort (*p* = 0.04). No statistical significance was found in FXI, PT or protein C, which had less data points available than AT (Fig. 1).

Consistent with the improvement in AT, no major thrombotic events were reported in any of the patients on D-gal. Three patients reported epistaxis, bruising or easy bleeding (P69, P71, P72). As no coagulation parameters were measured at the time, it is not possible to determine whether increased bleeding was linked to the disbalance in coagulation factors. Therefore, standardization of the baseline labwork in PGM1-CDG patients is necessary to provide additional data for futures studies as well as potentially identify individuals at risk for clinically significant events.

## 5. Conclusion

Coagulation factors are often abnormal in PGM1-CDG and major vascular events have been recorded in several patients. Nevertheless, little is known about the natural history of coagulation parameters in PGM1-CDG and the ability of D-gal to improve coagulation.

Here, we show that majority of the PGM1-CDG individuals present with coagulation abnormalities and that there is an increased risk of major vascular events in PGM1-CDG individuals, likely due to coagulation abnormalities. Moreover, in the patients that were longitudinally followed up in the FCDGC natural history study, we showed that those receiving D-gal treatment on average have a more normal biochemical coagulation profile. Specifically, longitudinally followed patients D-gal supplementation showed an improvement in coagulation factors, such as AT (Table 2, Fig. 1). While the three major (vascular) events were recorded in our cohort, none of the events happened during D-gal treatment. Hence, D-gal treatment seems to be protective against acute episodes related to abnormal hemostasis in PGM1-CDG.

Nevertheless, while we did note an overall improvement or normalization of coagulation parameters on D-gal, there was a lot of fluctuation between different time points, complicating the data analysis. In addition, some of the factors, such as FVII and FX were not frequently assessed in PGM1-CDG individuals, and no conclusions about their natural history or the effect of D-gal on them could be made. As AT was the most commonly assessed coagulation parameter, we could conclude that the average levels of AT improved on D-gal (Fig. 1) in a statistically significant way. Therefore, thorough clinical follow-up of other coagulation parameters prior to initiating D-gal therapy and during the treatment is necessary in order to conduct statistical assessment.

We conclude that AT could be used as a one of the clinical endpoints for future D-gal clinical trials. We urge clinicians to conduct a thorough assessment of both procoagulants and coagulation inhibitors in PGM1-CDG individuals in order to improve clinical care and establish better clinical endpoints for future clinical trials.

## Supplementary Material

Supplementary material

## Figures and Tables

**Table 1:** The breakdown of demographic data and coagulation parameters in previously reported PGM1-CDG individuals.

Previously reported PGM1-CDG individuals (*n* = 73)							
Gender						M = 44 (60.2 %). F = 29 (39.7 %)	
Age						Mean 12.7 (range 4mo-53 years)	
Coagulation evaluation						*N* = 43 (58.9 %)	
Coagulation abnormalities						*N* = 29 (67.4 %)	
D-Gal therapy						*N* = 19	
Coagulation parameters	Individuals with any information for the specific parameter (n)	Individuals reported without D-Gal (n)	Individuals with abnormal values without D-Gal (n)	Mean value	Individuals reported on D-Gal * (n)	Individuals with abnormal values on D-Gal (n)	Mean Value*
AT	10	9	9	30.8 % (*n* = 9) (R: 80–130 %)	7	5	64.5 % (R: 80–130 %)
FVII	1	1	1	15.8 % (R: 50–150 %)	0		
FIX	6	6	0	88 % (n = 2) (ref 50–200 %)	5	0	73 %(*n* = 2) (R: 50–200 %)
FX	1	1	1	45 % (R: 50–150 %)			
FXI	4	4	4	30.5 % (n = 4) (R 70–120 %)	2	2^$^	60.5 % (n = 2) (R: 70–120 %)
Protein C activity	3	3	3	33 % (n = 3)	0		
Protein S activity	3	3	3	41.66 % (n = 3) (R: 70–120)	0		
aPTT	11	11	8	41 s (n = 6) (R 24–45)	2	1	43.5 s (R 24–45) (n = 2)
PTT	1	1	1	41.8 s (R: 18–28 s)	0		
PT	7	7	6	15.1 s (n = 3) (R: 9–14)	1	1	17.2 (n = 1) (R 9–14)
Major vascular events		N = 4 (including DVT. death due to cerebrovascular event. and thrombosis)					

* if multiple measurements were reported, the lowest value recorded on D-Gal was used to calculate the average.

$ values normalized long term Remark: In several patients the actual values for parameters were not given in the publication, instead, the status of the parameters were described (e.g. abnormal, delayed).

**Table 2:** The evolution of coagulation parameters in longitudinally followed 16 PGM1-CDG individuals. Out of 16 individuals. 15 individuals received D-Gal. Coagulation abnormalities were reported in all 16 patients. Means for each coagulation parameter are given with and without D-Gal. Abnormal values are highlighted in red. Further demographic data of the individuals is presented in Supplementary Table 2. All the measurements at different time points and the reference ranges for each coagulation parameter are available in Supplementary Table 3.

Prospectively followed PGM1-CDG individuals (n=16)																		
Gender					**M=44 (60.2 %). F=29 (39.7 %)**													
Age					**Average 12.5 (range 4mo - 53 yo)**													
Coagulation evaluation					**N=16 (100 %)**													
Coagulation abnormalities					**N=16 (100 %)**													
D-Gal therapy					**N=15**													
Major (vascular) events without D-Gal					**N=3**													
Major (vascular) events on D-Gal					**N=0**													
**ID**	**P14**	**P67**	**P68**	**P69**	**P70**	**P71**	**P72**	**P73**	**P74**	**P75**	**P76**	**P77**	**P78**	**P79**	**P80**	**P81**		
**Currant age**	**23**	**32**	**6**	**29**	**5**	**4**	**31**	**29**	**2**	**3**	**23**	**15**	**6**	**9**	**4**	**19**		
**D-Gal time**	**>8y**	**>4 y**	**>3y**	**>4y**	**>2.5y**	**>3.5y**	**>6y**	**<1y**	**<1y**	**<1y**	**<2.5y**	**X**	**>3y**	**<1y**	**>2y**	**>3.5y**		
*Average, the lowest and the highest values for specific parameter per Individual (reference ranges In Sup Tab 3)*. *Abnormal reported values in red*																	*Mean*	Datapoints
**Platelet count**	426.9 (116–833)	NR	379.2 (92–635)	219.8 (164–283)	NR	NR	NR	NR	NR	NR	202.5 (173–271)	409.68 (161–833)	NR	326	371	228	321.02	64
**on D-Gal**	237.9 (199–284)	335.66 (307–375)	402.6 (118–691)	247.4 (219–295)	NR	330	216	281	NR	NR	196.5 (186–207)	NA	320.43 (195–437)	322 (319–325)	394.6 (250–712)	248.5 (226–271)	294.39	63
**AT III**	58.5 (37–80)	NR	28.1 (27.2–29)	NR	NR	23	NR	NR	57	102	68	64	NR	28.5 (26–31)	79 (58–98)	76	60.84	18
**on D-Gal**	98 (74–131)	62.9 (51–83)	70.2 (44–146)	130	46	85.1 (60–110)	78.6 (67–93)	41	92.5 (80–105)	80	69 (59–79)	NA	75.14 (48–111)	78 (56–100)	83	88 (84–92)	78.70	70
**Factor VII (FVII)**	56	NR	NR	NR	NR	NR	NR	NR	NR	76	NR	NA	NR	26.6	374.2	NR	133.2	4
**on D-Gal**	NR	NR	39 (23–59)	NR	NR	NR	NR	NR	NR	NR	NR	NA	NR	NR	NR	NR	39	3
**Factor IX (FIX)**	0.74	0.83	0.64	NR	NR	0.32	NR	NR	NR	0.61	NR	NA	NR	NR	1.45	NR	0.74	6
**on D-Gal**	0.86 (0.74–1.15)	NR	0.618 (0.33–0.85)	NR	0.72	0.71 (0.52–1.02)	NR	NR	0.64	0.69	0.57	NA	NR	NR	NR	NR	0.71	21
**Factor X (FX)**	102	NR	NR	NR	NR	NR	NR	NR	NR	72	NR	NA	NR	62	NR	NR	78.7	3
**on D-Gal**	NR	NR	NR	NR	NR	NR	NR	NR	67	86	NR	NA	NR	NR	NR	NR	76.5	2
**Factor XI (FXI)**	0.53 (0.44–0.62)	NR	0.135 (0.13–0.14)	NR	NR	0.06	NR	NR	NR	0.79	NR	0.53	NR	0.176 (0.16–0.19)	1.125	NR	0.48	10
**on D-Gal**	0.81 (0.78–1.39)	1.02 (0.76–1.38)	0.414 (0.24–0.73)	NR	0.73 (0.58–0.88)	0.52 (0.36–0.8)	0.58 (0.48–0.69)	0.41	0.43	0.58	0.67 (0.5–0.83)	NA	0.583 (0.58–0.59)	0.70	0.503 (0.34–0.71)	NR	0.61	44
**Protein C activity**	0.573 (0.41–0.91)	NR	0.28	1.33	NR	NR	NR	0.76 (0.55–0.94)	0.36	0.66	0.55	NA	NR	0.15	0.41	NR	0.56	15
**on D-Gal**	NR	NR	0.38 (0.1–0.64)	NR	NR	0.59 (0.56–0.63)	NR	0.58	0.6	0.89	NR	NA	NR	NR	0.32	NR	0.56	11
**Protein S activity**	48 (34–60)	NR	NR	NR	NR	NR	NR	NR	NR	78.8	NR	NA	NR	50	111	NR	71.95	9
**on D-Gal**	NR	NR	NR	NR	NR	96	NR	NR	56 (45–67)	99	55	NA	NR	NR	50	NR	71.2	6
**aPTT**	NR	27 (27)	34.9 (32.5–38)	26.8 (23–32)	NR	41.6	31.3	Normal	NR	NR	NR	28.25 (22–31)	NR	NR	NR	NR	31.64	14
**on D-Gal**	NR	NR	36	41.33 (36–48)	NR	32.5 (30.3–34.4)	26.3 (24.9–27.8)	29	30	NR	NR	NA	NR	NR	NR	25	31.44	20
**PTT**	32.9 (27.3–43)	11.4 (10.5–12.6)	NR	NR	NR	NR	NR	27.8 (24.7 – 31)	NR	32.4	29.15 (28–30.3)	30.72 (26.6–36)	NR	31.5	24.4	NR	27.53	21
**on D-Gal**	27.9 (24–29.7)	NR	30.87 (23.5–37.2)	NR	28	NR	NR	NR	NR	26	31.15 (30.1–32.2)	NA	26.37 (19.2–30.5)	28.55 (25.6–31.5)	42.42 (33.9–51.5)	NR	30.15	29
**PT**	13.5 (12–17.4)	NR	11.73 (10.6–12.7)	18.1	NR	15.2 (14.3–16.5)	9.7	15	11	12.7	13.35 (12.7–14)	10.66 (9.9–11.6)	NR	13	14.3	NR	13.19	35
**on D-Gal**	12.66 (11.7–14.5)	NR	15.69 (13.6–18.2)	NR	NR	14.3 (13.9–14.7)	10.6 (10.4–10.9)	NR	11	16.2	12.85 (12.7–13)	NA	13.23 (11.4–14.7)	12.9 (12.1–13.7)	32.21 (19–88)	NR	16.88	68
**Total severity score** mild (0–14). moderate (15–25) and severe (>26)	NR	NR	18	NR	NR	11	NR	5	NR	7	3	17	NR	NR	NR	NR	10.16	7
**On D-Gal**	3.75 (2–7)	6.5 (6–9)	14 (7–20)	11 (6–13)	8.5 (7–10)	7.5 (5–11)	7	NR	21.7 (21–23)	5	3	NR	NA	19.2	NR	NR	8.83	36

## Data Availability

No data was used for the research described in the article.

## Appendix A. Supplementary data

Supplementary data to this article can be found online at https://doi.org/10.1016/j.ymgme.2024.108530.

## Abbreviations:

aPTT: activated partial thromboplastin time
AT: Antithrombin
CDG: Congenital Disorder(s) of Glycosylation
D-gal: D-galactose treatment
DVT: Deep Vein Thrombosis
FVII: Factor VII
FIX: Factor IX
FX: Factor X
FXI: Factor XI
MCA: Middle Cerebral Artery
NPCRS: Nijmegen Pediatric CDG Rating Scale
P: Patient
PGM1: Phosphoglucomutase-1
PMM2: Phosphomannomutase-2
PT: Prothrombin time
PTT: partial thromboplastin time
R: reference range
